# FineTea: A Novel Fine-Grained Action Recognition Video Dataset for Tea Ceremony Actions

**DOI:** 10.3390/jimaging10090216

**Published:** 2024-08-31

**Authors:** Changwei Ouyang, Yun Yi, Hanli Wang, Jin Zhou, Tao Tian

**Affiliations:** 1 School of Mathematics and Computer Science, Gannan Normal University, Ganzhou 341000, China; 2 Department of Computer Science and Technology, Tongji University, Shanghai 201804, China; hanliwang@tongji.edu.cn; 3 School of Computer Science and Artificial Intelligence, Chaohu University, Hefei 238024, China

**Keywords:** tea ceremony actions, fine-grained action recognition, temporal shift module, ConvNeXt

## Abstract

Methods based on deep learning have achieved great success in the field of video action recognition. When these methods are applied to real-world scenarios that require fine-grained analysis of actions, such as being tested on a tea ceremony, limitations may arise. To promote the development of fine-grained action recognition, a fine-grained video action dataset is constructed by collecting videos of tea ceremony actions. This dataset includes 2745 video clips. By using a hierarchical fine-grained action classification approach, these clips are divided into 9 basic action classes and 31 fine-grained action subclasses. To better establish a fine-grained temporal model for tea ceremony actions, a method named TSM-ConvNeXt is proposed that integrates a TSM into the high-performance convolutional neural network ConvNeXt. Compared to a baseline method using ResNet50, the experimental performance of TSM-ConvNeXt is improved by 7.31%. Furthermore, compared with the state-of-the-art methods for action recognition on the FineTea and Diving48 datasets, the proposed approach achieves the best experimental results. The FineTea dataset is publicly available.

## 1. Introduction

Video content analysis is a significant research problem in the field of computer vision with widespread applications in intelligent surveillance, healthcare, human–computer interaction, etc. [1]. Action recognition is one of the basic tasks of video content analysis and aims to recognize different human activities or action categories in videos. Fine-grained action recognition is an important subfield of action recognition. It focuses on identifying fine-grained actions from coarse-grained activities and includes research tasks from various coarse-grained activities, e.g., cooking [2], baseball [3], diving [4], table tennis [5], etc. The tea ceremony has profound cultural connotations and elegant fine-grained actions, which bring new challenges to fine-grained action recognition. Tea ceremony action recognition is a new task in the research area of fine-grained action recognition, opening up new application scenarios for this research area. In general, tea ceremony action recognition has a wide range of applications, e.g., tea ceremony robots, smart education about the tea ceremony, promotion of tea culture, etc. These applications of the research results on tea ceremony action recognition can enrich cultural experiences, improve operational efficiency, and foster connections between people and their cultural heritage.

In recent years, with the vigorous development of deep learning models [6,7,8], the field of video action recognition has made significant progress and breakthroughs. Although these methods have made impressive progress on datasets like HMDB51 [9], UCF101 [10], and Kinetics-400 [11], they only address a portion of the challenges in the field of action recognition. One limiting factor is that most existing action recognition datasets primarily focus on coarse-grained action categories, e.g., playing basketball, making tea, etc. This causes a gap for many practical applications that require fine-grained action analysis.

**Figure 1:**
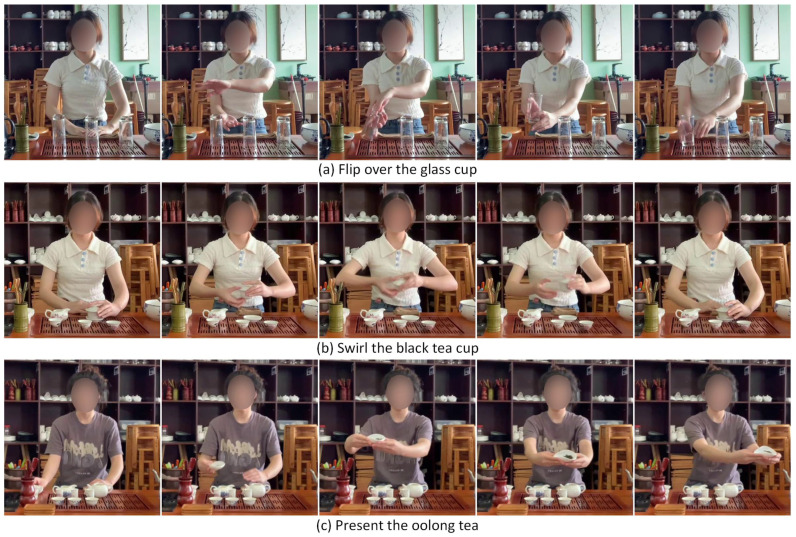
Examples of tea ceremony actions with high similarity.

As shown in Figure 1, different tea ceremony actions show high similarity. In general, tea ceremony action recognition confronts two primary challenges, i.e., low inter-class variation and strong temporal dependencies. Specifically, there is low variation between the classes of tea ceremony actions, as the differences between these actions are mainly reflected in the interactions between hands and tea utensils. Moreover, the occurrence sequence, duration, and transitions between tea ceremony actions are closely related in the temporal domain. As shown in Section 5.6, these two challenges make the experimental performance of existing methods relatively low. In addition, there is a lack of publicly available datasets for tea ceremony action recognition, which hinders research in the field of tea ceremony action recognition.

To address the above issues, a fine-grained video dataset for the task of tea ceremony action recognition is constructed, which is named Fine-grained Tea (FineTea). To the best of our knowledge, FineTea is the first video dataset specifically designed for the fine-grained recognition task of tea ceremony actions. Unlike existing datasets, the FineTea dataset is hierarchically subdivided into tea ceremony activities based on verb attributes, and it encompasses a large number of videos and diverse action categories. This dataset comprises 2745 video clips. By using a hierarchical fine-grained action classification method, these video clips are categorized into 9 basic action classes and 31 fine-grained action subclasses. Additionally, the action features of participants performing the same type of tea ceremony activity are highly consistent across different video clips. This consistency makes it challenging to assess the generalization ability of models using the conventional random split of training and testing datasets. Therefore, a non-repeating occurrence algorithm based on dynamic programming is designed that divides the dataset into two sets at a ratio of about 50%: namely, set 1 and set 2. To mitigate potential biases caused by a particular split of the data, a two-fold cross-validation strategy is employed in our experiments. Consequently, two splits of the dataset are obtained: denoted as split 1 and split 2. In split 1, set 1 is used for training, and set 2 is used for testing. Conversely, in split 2, set 2 is utilized for training, and set 1 is utilized for testing. So the percentages of training and testing data are about 50% each. Four state-of-the-art action recognition methods [12,13,14,15] are evaluated on the FineTea dataset, and the experimental results demonstrate that the dataset is challenging.

Based on the ConvNeXt [16] architecture, a fine-grained action recognition method named TSM-ConvNeXt is proposed as a benchmark method for the FineTea dataset. TSM-ConvNeXt achieves strong capabilities for spatiotemporal modeling by employing a high-performance convolutional neural network as the model backbone and introducing a temporal shift module (TSM) [17] to model temporal information in videos. On the FineTea dataset, TSM-ConvNeXt obtains a 7.31% improvement over the baseline TSM method [17]. Compared with the state-of-the-art action recognition methods, the proposed TSM-ConvNeXt achieves the best experimental results on the FineTea and Diving48 datasets. The main contributions are summarized as follows.

1. FineTea is built as the benchmark dataset for the task of fine-grained action recognition and includes high-quality and fine-grained annotations. The FineTea dataset is publicly available at https://github.com/Changwei-Ouyang/FineTea (accessed on 27 August 2024).
2. For fine-grained action recognition, the TSM-ConvNeXt network is designed to enhance temporal modeling capability. The proposed TSM-ConvNeXt achieves better performance than the baseline methods. Moreover, TSM-ConvNeXt obtains the best experimental results on the FineTea and Diving48 datasets.

## 2. Related Work

### 2.1. Coarse-Grained Action Recognition Datasets

Datasets are essential for the success of deep learning methods, so researchers are working hard to create higher-quality datasets. Although each dataset has its specific motivation, their common goal is to provide a unified evaluation standard, which in turn promotes the development of the related fields. Early datasets can be traced back to KTH [18] and Weizmann [19], followed by more challenging datasets such as HMDB51, UCF101, Kinetics, ActivityNet [20], and a series of other datasets [21,22]. Although these datasets have made significant progress in providing class labels, they are still limited to coarse-grained action categories, such as playing basketball or making tea. In coarse-grained datasets, neural network models often learn features that are not the action itself but the background context, which may lead to the network model’s lack of focus on the action itself. So the generalization ability of the model is reduced. In [23], the TSN model achieved an accuracy of 85% on the UCF101 dataset using only three frames in training. This suggests that models are easily affected by the learning of background features when using coarse-grained datasets for video action recognition. Therefore, there is an urgent need to create more challenging fine-grained datasets to further promote research and development in the field of action recognition.

### 2.2. Fine-Grained Action Recognition Datasets

To enable action recognition methods to focus more on action motion and temporal context information, researchers have tried to construct datasets for fine-grained action recognition. For example, Rohrbach et al. [2] annotated the individual steps of various cooking behaviors, defined the verb part of the action as the fine-grained action category, such as “cut” for the action of cutting carrots, and constructed the MPII Cooking dataset. Goyal et al. [24] collected everyday human–object interactions, such as putting down objects or picking up objects from somewhere, and built the Something–Something V1 dataset. Based on the combination of four action attributes, Li et al. [4] collected 48 fine-grained diving action videos and established the Diving48 dataset. Shao et al. [25] focused on collecting fine-grained action videos from gymnastics competitions and constructed two versions of fine-grained action recognition datasets for gymnastics. Piergiovanni et al. [3] constructed the MLB-YouTube dataset by collecting videos of various fine-grained baseball actions such as hit, strike, swing, and foul. Martin et al. [5] recorded videos of table tennis matches between 17 different players in real-world environments, annotated fine-grained table tennis actions such as the serve forehand sidespin and the serve backhand backspin, and built a fine-grained table tennis action recognition dataset.

A comprehensive comparison between the proposed FineTea dataset and other fine-grained action recognition datasets is presented in Table 1. In this table, “Number of classes” refers to the number of action categories in the corresponding dataset. “Number of clips” indicates the number of video clips in each dataset. “Video source” represents the origin of the video clips: for example, “Self-collected” means that the videos were recorded by the creators of the dataset, while “Major League Baseball” and “Diving competition” indicate that the videos were obtained from baseball and diving events, respectively. In contrast to these datasets, FineTea has the following characteristics. First, FineTea contains more complex hand gestures, which causes new challenges for action recognition methods. Second, FineTea adopts a unique non-repeating appearance split strategy to ensure that participants’ videos in the same action category do not appear in both the training set and testing set simultaneously. Third, based on expert knowledge, a unified standard is applied to annotate all categories, and cross-checking is carried out to ensure the accuracy and consistency of annotations.

### 2.3. Action Recognition Methods

The task of video action recognition aims to recognize human actions in a video. Due to its wide range of applications in the real world, researchers have explored this area extensively in the past few decades, with techniques developing from early hand-crafted feature methods to deep learning models [26,27]. Early attempts included DeepVideo [28], which was the pioneering work that first applied convolutional neural networks to video content understanding. Subsequently, the Two-Stream Network [29] started a new direction by training convolutional neural networks on the optical flow stream as a second path to capture the temporal information in videos. The Two-Stream Network method surpassed traditional hand-crafted feature methods and established the groundwork for subsequent research. Based on this study, many innovative methods have emerged, such as Fusion [30], TSN [23], TSM [17], etc. However, optical-flow-based methods have the problem of high computational cost, which prompted researchers to design 3D convolutional structures to better capture temporal features and semantic information in videos; examples include I3D [31], Non-local [32], and SlowFast [33]. With the success of the Transformer model, which is based on a self-attention mechanism, in the fields of natural language processing and image recognition, researchers in the field of video action recognition have started to explore the application and development of Vision Transformers (ViTs) [34]. TimeSformer [12] extended the self-attention mechanism to the temporal dimension by incorporating spatiotemporal modules into the ViT model so that the model can handle spatiotemporal information in videos. Currently, methods based on Transformer structures have achieved state-of-the-art results on most video action recognition datasets.

## 3. FineTea Dataset

**Figure 2:**
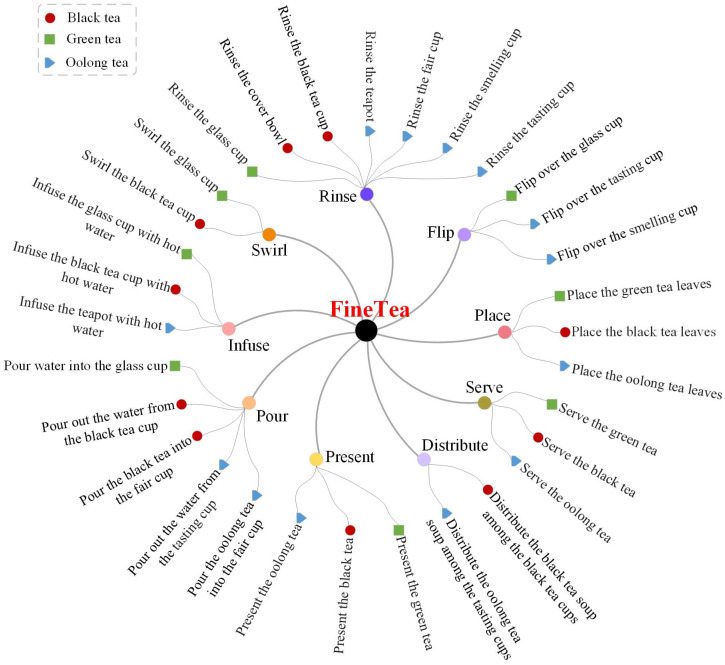
An overview of the actions in the FineTea dataset.

The goal of the FineTea dataset is to create a challenging benchmark with high-quality annotations and with a particular focus on tea ceremony actions. This dataset includes three types of tea ceremony activities, i.e., the green tea activity, the black tea activity, and the oolong tea activity. The green tea activity consists of 8 fine-grained actions that require participants to use a glass cup to brew green tea with relatively simple steps. The black tea activity consists of 10 fine-grained actions that require participants to brew black tea using the bowl-covering method. The oolong tea activity involves 13 fine-grained actions and requires participants to use the double-cup technique to brew oolong tea. In total, the FineTea dataset includes 3 types of tea ceremony activities, 9 basic actions, and 31 fine-grained actions, as shown in Figure 2.

### 3.1. Video Collection

In the process of collecting tea ceremony action videos, a total of 18 volunteers participated for the recording of the videos. These volunteers completed a tea ceremony course, and their tea ceremony skills range from a beginner level to that of amateur tea artists. This varying skill level among the volunteers enables the FineTea dataset to incorporate actions spanning different skill levels for the tea ceremony, thereby making it a particularly challenging dataset. The process includes three categories of tea ceremony activities, i.e., brewing green tea, brewing black tea, and brewing oolong tea. Before starting the process, these participants were instructed on examples of tea ceremony actions. This ensured that they could accurately perform and complete each tea ceremony action.

The recording process followed a strict procedural flow to ensure the accuracy and consistency of data collection. The experimental paradigm is described as follows. First, a volunteer places the phone stand 1 m in front of the participant and adjusts the angle to ensure that all action details appear completely in the video. Second, to precisely capture valid action data, the recording begins after the participant takes a seat at the tea table and makes a ready gesture, and recording ends when the “serve tea” action is completed and excludes the last tea-tasting session. Third, the entire recording time for the tea ceremony activities is approximately 4 to 9 min. Each participant conducts several sessions for the tea ceremony action collection.

Altogether, the participants generated a total of 272 long videos covering three types of tea ceremony activities. The total duration of these videos exceeds 1185 min, which is equivalent to about 2,133,000 frames. These videos were recorded in a tea ceremony classroom. The resolution of these videos is 1280×720 pixels, and the frame rate is 30 frames per second.

### 3.2. Annotation

After completing the process of video recording, a series of steps were taken to annotate these videos. According to the type of tea (i.e., green tea, black tea, or oolong tea), we put the 272 long videos into three tea-level folders, each of which includes videos about a specific tea ceremony activity. Then, videos in each tea-level folder were annotated in detail according to the established fine-grained actions in the corresponding tea ceremony activity. After annotating all of the long videos, we obtained a total of 2745 video clips. Based on the results of the annotations, these video clips were divided into 31 fine-grained actions.

Table 2 reports the statistics of the FineTea dataset. As shown in this table, the number of clips for each fine-grained action may be different. The main reasons for this phenomenon are as follows. First, some fine-grained actions are repeated twice in a tea ceremony activity, e.g., “Infuse the black teacup with hot water”, “Pour the black tea into the fair cup”, etc. Second, some video clips were discarded because the volunteers’ fine-grained actions in these clips did not meet the standards of the corresponding tea ceremony activity.

To ensure high-quality annotations of tea ceremony actions in each video, two specially trained annotators were invited. They were familiar with the annotation standards and methods for fine-grained action categories. The annotation tasks were to determine the starting frame and the ending frame of each action instance in the videos and to assign the corresponding action labels. The EIVideo tool (https://github.com/PaddlePaddle/PaddleVideo/tree/develop/applications/EIVideo, accessed on 27 August 2024) was utilized to annotate these videos; it is an interactive intelligent video annotation toolbox provided by the Baidu PaddlePaddle platform. By using this tool, the annotators rapidly previewed the video content, precisely located the starting and ending frames of the action instances in the video timeline, and assigned the respective action labels. This interactive strategy not only improves annotation precision but also reduces the potential for mislabeling and the time required. To ensure the accuracy and consistency of the annotation results, the two annotators cross-checked each other’s annotation results.

### 3.3. Dataset Split

After analyzing the characteristics of tea ceremony actions in the dataset, a common trend is observed: that is, the action characteristics of participants exhibit high consistency when they perform the same type of tea ceremony actions in different video clips. This raises a crucial concern that neural network models might tend to take shortcuts during the learning process and rely on features unrelated to the actions themselves. These features may be contextually irrelevant background information such as clothing or appearance in videos. This could potentially impact the generalization ability of networks across different environments.

To better evaluate the generalization performance of network models, it is crucial to ensure that video clips of the same participant performing the same tea ceremony action do not simultaneously appear in both the training set and the testing set. Therefore, a non-repetition algorithm based on dynamic programming for dataset splitting was designed to implement two types of splits. The algorithm includes the following steps. First, for each category of tea ceremony fine-grained actions, all data are aggregated in the action-level folders, each of which contains videos about a specific fine-grained action. The number of participants and the number of their corresponding video clips are calculated. Second, the video clips of each participant are regarded as an indivisible entity, and a dynamic programming algorithm is utilized to combine videos with different participants at a ratio of about 50%. Third, based on the partitioning results, these videos are assigned to either the training set or the testing set.

Therefore, two distinct sets are generated for model training and testing. Table 3 shows the statistics of the two sets. A two-fold cross-validation strategy is employed to obtain two distinct splits, denoted as split 1 and split 2. In split 1, set 1 is used for training and set 2 is used for testing. Conversely, in split 2, set 2 is used for training and set 1 is used for testing.

## 4. Proposed Method

In this section, the proposed method for action recognition is detailed. First, the backbone network ConvNeXt is introduced in Section 4.1. Following that, Section 4.2 presents the temporal shift module. Finally, the network architecture of the TSM-ConvNeXt model is illustrated Section 4.3.

### 4.1. ConvNeXt Backbone

**Figure 3:**
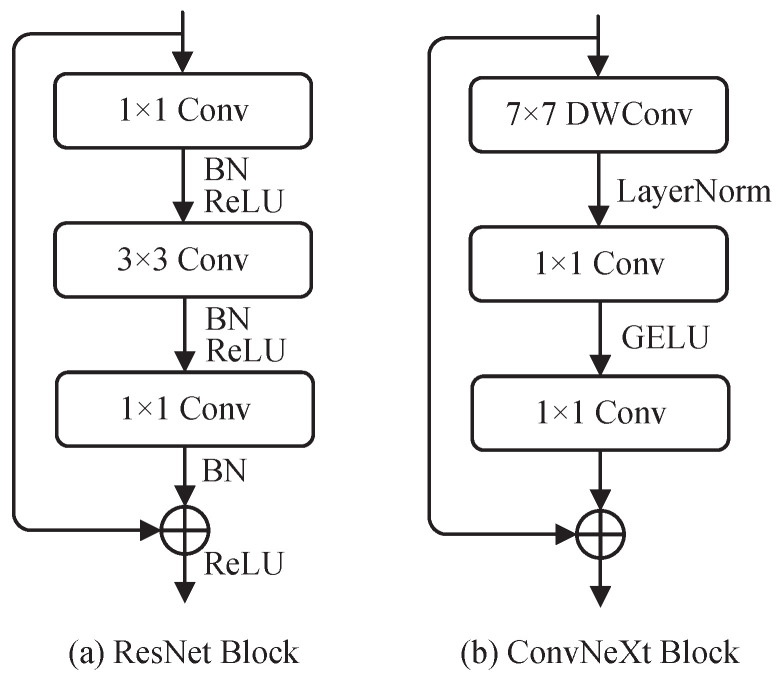
A structural diagram of the ResNet [6] block and ConvNeXt [16] block.

The ConvNeXt network is an efficient convolutional neural network with a structural design similar to the Swin Transformer network [35] and ResNet [6]. To introduce the ConvNeXt block more clearly, Figure 3 shows a comparison between the ResNet Block and ConvNeXt Block. As shown in this figure, compared to the traditional ResNet block, the ConvNeXt block utilizes depth-wise convolution (DWConv) with larger kernels. Additionally, it uses fewer regularization and activation functions, replacing the Batch Normalization (BN) layer [36] and ReLU with a LayerNorm layer [37] and GELU, respectively. This design transforms the architecture of ConvNeXt into a convolutional neural network with the style of a Transformer.

In order to adapt to different tasks, the ConvNeXt network provides various variants with different scales. Among them, ConvNeXt-T, ConvNeXt-S, ConvNeXt-B, and ConvNeXt-L respectively correspond to Swin-T, Swin-S, Swin-B, and Swin-L in the Swin Transformer network. According to the experimental results in [16], ConvNeXt-B achieves a recognition accuracy of 83.8% on the ImageNet dataset [38], and the performance of the corresponding ConvNeXt models surpasses that of Swin Transformer networks. With its concise and efficient structural design and outstanding performance, ConvNeXt demonstrates significant potential for applications in the field of video content analysis.

### 4.2. Temporal Shift Module

The temporal shift module (TSM) is a critical component designed specifically for video content analysis [17]. The primary purpose of the TSM is to capture temporal information within video sequences. The TSM achieves this by performing shift operations on input features along the temporal dimension. This introduces variations in the temporal domain. So the spatiotemporal model of a video can be built by integrating the TSM into two-dimensional convolutional neural networks (2D CNNs).

When applied to the fine-grained action recognition task for tea ceremony actions, the TSM introduces temporal variations between adjacent frames by using the bidirectional channel shifts in the 2D CNNs. This enables the establishment of a model with specific time information for tea ceremony actions.

### 4.3. TSM-ConvNeXt

**Figure 4:**
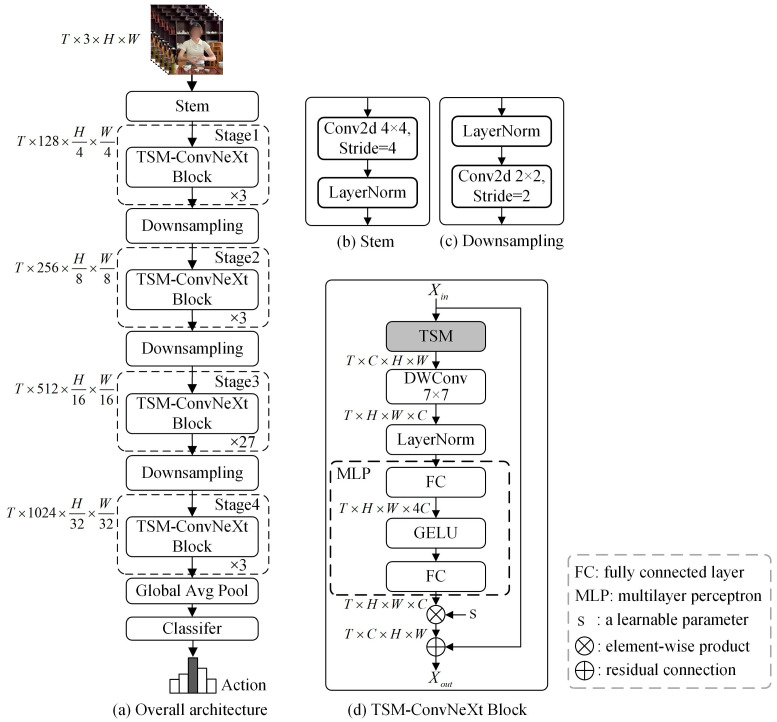
The overall hierarchical architecture of TSM-ConvNeXt.

To establish a fine-grained temporal model for tea ceremony actions, a fine-grained action recognition method based on the ConvNeXt network, named TSM-ConvNeXt, is proposed as the benchmark method for the FineTea dataset. By using the high-performance ConvNeXt-B as the backbone and introducing the TSM to model temporal information in videos, the TSM-ConvNeXt method can build a stronger spatiotemporal model with lower computational cost. The overall framework of the network is depicted in Figure 4. The sampled video frames are input into a 2D convolution layer with a 4 × 4 kernel. There are four stages in TSM-ConvNeXt. The number of blocks stacked in each stage is 3, 3, 27, and 3, respectively. Between two consecutive stages, a downsampling module is utilized to double the number of channels (i.e., *C*) and reduce the spatial dimensions (i.e., *H* and *W*) by half. As shown in Figure 4c, the downsampling module includes a LayerNorm layer and a convolutional layer with a kernel size of 2×2 and a stride of 2. The predictions of action categories are obtained by using a global average pooling layer and a classification layer.

According to [17], directly inserting the TSM into the model may shift some channels to adjacent frames, which renders the feature information in those channels unavailable for the current frame and severely compromises the spatial feature learning ability of the model. Inspired by [17], a strategy is employed to shift only a portion of the channels. The degree of channel shifting is controlled by a shift proportion parameter. Considering the residual connection block structure of the ConvNeXt block, the TSM block is placed before the DWConv layer in the ConvNeXt block.

The TSM-ConvNeXt block contains a TSM block, a DWConv layer with a 7×7 kernel, a LayerNorm layer, and a MultiLayer Perceptron (MLP) module. The MLP module consists of a fully connected (FC) layer for upsampling, a GELU activation function, and an FC layer for downsampling. Let $X_{in}\in R^{T\times C\times H\times W}$ be the input tensor, where *T* is the number of sampled video frames, *H* and *W* are the height and width, respectively, and *C* is the number of feature channels. After the DWConv layer, the tensor’s dimensions are permuted to T×H×W×C, which allows the fully connected layer to operate along the *C* dimension. Before the residual connection, the dimensions of the tensor are rearranged back to T×C×H×W, thereby ensuring consistency between the input and output dimensions of the module. The output tensor after the ConvNeXt block $X_{out}\in R^{T\times C\times H\times W}$ can be obtained as follows.

(1) $$X_{1}=\mathrm{LayerNorm}\left(\mathrm{DWConv}\left(\mathrm{TSM}\left(X_{in}\right)\right)\right)$$

(2) $$X_{out}=X_{in}+s⊗\mathrm{MLP}\left(X_{1}\right)$$

where LayerNorm(·) is the function of the LayerNorm layer, DWConv(·) is the function of the DWConv layer, TSM(·) is the function of the TSM block, ⊗ is the element-wise product, $s\in R^{C}$ is a learnable parameter for scaling the feature map, and MLP is the function of the MLP block.

## 5. Experiment

### 5.1. Dataset

To evaluate the proposed method, extensive experiments were conducted on two fine-grained action recognition datasets: FineTea and Diving48 [4]. The FineTea dataset was introduced in Section 3. Regarding the evaluation, the performances of the different methods on this dataset were evaluated by using a two-fold cross-validation strategy. The average recognition accuracy was obtained by averaging the experimental results of the two splits, and it was used for performance comparisons between different methods. The Diving48 dataset is a large-scale fine-grained action recognition dataset containing 48 diving action categories. According to the standard experimental process [4], we used approximately 15 k videos to train networks and 2 k videos to test the trained models, and we reported the Top-1 accuracy.

### 5.2. Experimental Setup

The pre-trained weights of ConvNeXt on the ImageNet dataset are used as the initial weights of the model. Then, the parameters are optimized to effectively reduce the training time, and the performance of the model is improved for the task of fine-grained action recognition. RGB frames are used as the input of the model.

In the experiments, the video data require a series of preprocessing steps before being input into the network for training. The video frames are sampled by using a sparse sampling strategy. Specifically, for an input video, the video is divided into *K* segments on average, and then one frame is randomly sampled from each segment of the video. Regarding the training of the network, the data augmentation strategy consistent with the TSM method [17] is adopted. Specifically, the frame is first cropped to a width of 256 pixels, and then the size is adjusted to 224×224 pixels using multi-scale cropping and center cropping strategies. A flipping operation with a probability of 0.5 is performed. The SGD optimizer is used to train the network model. The learning rate is set to 0.01, the weight decay is 1 × 10^−4^, the momentum parameter is 0.9, and the learning rate is reduced ten times in the twentieth and fortieth epochs. The total number of epochs is 50. For the FineTea dataset, 8 frames are uniformly sampled, and the batch size is fixed to 8. Regarding the Diving48 dataset, 32 frames are uniformly sampled, and the batch size is set to 4. In the testing stage, only the center cropping strategy is performed.

### 5.3. Comparison with the Baseline Method

In this section, a comparison experiment is conducted to verify the effectiveness of the TSM-ConvNeXt method. The TSM method [17] with ResNet50 as the backbone network is selected as the baseline method. The pre-trained weights on the ImageNet dataset are used as the initial weights for both methods. To ensure a fair comparison, these methods utilize the same hyperparameters as introduced in Section 5.2. Moreover, the shift proportion parameter of TSM-ConvNeXt is empirically set to 1/8.

**Figure 5:**
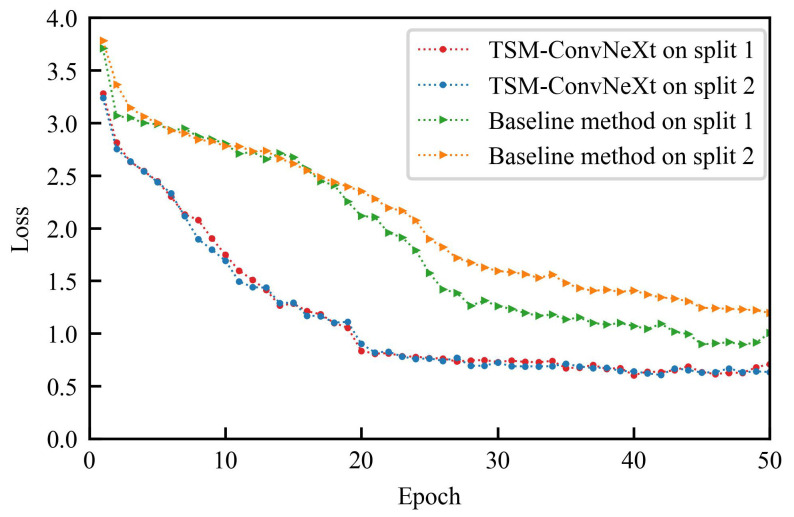
Comparison of training loss curves.

These methods are trained on the two splits of the FineTea dataset, i.e., split 1 and split 2. The loss curves are shown in Figure 5, where the horizontal axis is the number of epochs and the vertical axis is the loss value. As shown in this figure, TSM-ConvNeXt converges faster than the baseline method and reaches a lower loss value at convergence.

The experimental results are shown in Table 4. Compared to the baseline method, the results of TSM-ConvNeXt are improved by 4.53% and 10.09% for split 1 and split 2, respectively. The average recognition accuracy is improved by 7.31%. This demonstrates that TSM-ConvNeXt obtains a significant performance improvement over the baseline method for the task of fine-grained action recognition of tea ceremony actions. Moreover, a superior backbone can give the model a stronger temporal representation learning ability, thereby improving the performance of the model in downstream tasks.

### 5.4. Selection of the Hyperparameter

As introduced in Section 4.3, the shift proportion parameter has a critical impact on the performance of the model, as it controls the amount of channel shift in the temporal dimension of the TSM. To find the appropriate value of this parameter, ablation experiments are performed. The shift proportion parameter is set to 1/8, 1/4, or 1/2. The results on the FineTea dataset are shown in Table 5. For a fair comparison, only the shift proportion parameter is changed in these experiments; the other parameters for these three methods remain unchanged.

As shown in Table 5, the TSM-ConvNeXt method achieves the best experimental results on both split 1 and split 2 when this parameter is set to 1/4. This indicates that when this parameter is set too high, too much information is lost for the current video frame, which can damage the spatial feature learning ability of the network. Based on the analysis of the above results, when this parameter is set to 1/4, the model can better learn the temporal features of fine-grained actions in tea ceremony actions. Therefore, this parameter is fixed to 1/4 in the next experiments.

### 5.5. Ablation Experiments

To further validate the effectiveness of the proposed TSM-ConvNeXt method, we conducted ablation experiments on the FineTea dataset. The experimental results are shown in Table 6, where ”ConvNeXt” is the method without the TSM module. To make a fair comparison, all experiments in this section follow the same experimental setup.

As shown in this table, the TSM-ConvNeXt method obtains better experimental results than ConvNeXt on both split 1 and split 2 of the FineTea dataset. The experimental results partly demonstrate that incorporating the TSM enhances the temporal modeling capabilities of the network, thereby improving its performance in fine-grained action recognition tasks.

### 5.6. Comparison with State-of-the-Art Methods

#### 5.6.1. Comparison on the FineTea Dataset

In this section, four state-of-the-art action recognition methods (i.e., TimeSformer [12], VideoSwin [13], VideoMAE [14], and AIM [15]) are used to test the performance of fine-grained action recognition on the FineTea dataset. To compare in a relatively fair way, backbone networks with parameters equivalent to those of ConvNeXt-B are selected for these methods: namely, ViT-Base [34] and Swin-Base [35]. For the TimeSformer method, the space-only model [12] is selected for testing.

Regarding implementation, the MMAction toolbox [39] is utilized to carry out the experiments using TimeSformer and VideoSwin, and their official codes are used to conduct the experiments using VideoMAE and AIM. For TimeSformer, VideoSwin, and AIM, the training epochs are uniformly set to 50. VideoMAE is a self-supervised learning method, which usually requires more epochs to train the model. So the training epochs for VideoMAE are set to 300. As shown in Table 7, the parameters of the four methods are set according to the original settings. During the testing stage, all methods do not perform additional operations except for sampling the specified video frames.

The experimental results are shown in Table 8, where “TSM-ConvNeXt8” indicates the TSM-ConvNeXt method that samples 8 frames as model input, and “TSM-ConvNeXt16” is the TSM-ConvNeXt method that samples 16 frames. As shown in this table, among the four methods, TimeSformer obtains better experimental result than the others on split 1, AIM obtains the best result on split 2, and VideoSwin achieves the best average recognition accuracy. Compared with TimeSformer, which also samples 8 frames, TSM-ConvNeXt8 improves the average recognition accuracy by 2.9%. Compared with VideoSwin, which has the best performance among the four methods, the average accuracy of TSM-ConvNeXt8 is 2.82% higher than that of VideoSwin. Moreover, the performance of TSM-ConvNeXt is further improved when the method samples 16 frames. In summary, the proposed TSM-ConvNeXt obtains the best experimental results for the fine-grained action recognition task of tea ceremony actions.

#### 5.6.2. Comparison on the Diving48 Dataset

To further validate the effectiveness of the proposed TSM-ConvNeXt method, experiments were conducted on the Diving48 dataset. The experimental results are shown in Table 9. On the Diving48 dataset, the proposed TSM-ConvNeXt method achieves higher recognition accuracy than other CNN-based methods. Compared to the Transformer-based methods that use the same pre-trained weights, the proposed TSM-ConvNeXt method also obtains better performance than TimeSformer-L and RPE-STDT. In conclusion, TSM-ConvNeXt achieves better experimental performance than the other methods on the Diving48 dataset.

### 5.7. Comparison of Training and Testing Times

To evaluate the efficiencies of different methods, we conducted experiments on split 1 of the FineTea dataset. Table 10 reports the training and testing times of five methods, i.e., TimeSformer, VideoSwin, AIM, TSM-ConvNeXt8, and TSM-ConvNeXt16. Note that in this table, the training time is measured in hours, and the testing time is measured in seconds. For a fair comparison, all methods are trained for 50 epochs, and only one NVIDIA Tesla V100 GPU is used. As shown in this table, AIM has the lowest training time because it has the fewest tunable parameters [15]. Although the proposed TSM-ConvNeXt16 method requires longer training and testing times than AIM, it obtains a significant performance improvement. In conclusion, TSM-ConvNeXt achieves a balance between time cost and accuracy.

### 5.8. Analysis and Discussion

**Figure 6:**
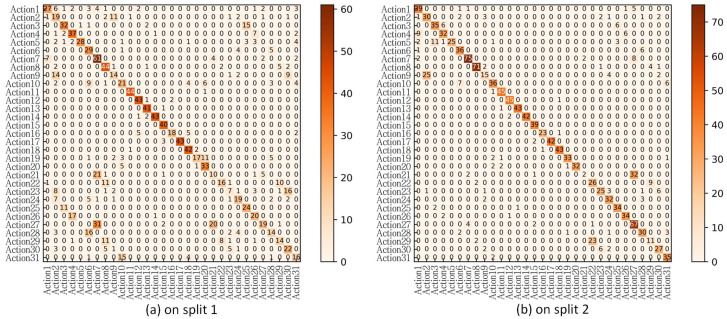
Confusion matrices for the test sets of the two splits.

To further analyze the fine-grained recognition of tea ceremony actions, the confusion matrices of the proposed TSM-ConvNeXt16 on split 1 and split 2 are shown in Figure 6. The figure visualizes the recognition results of the 31 actions contained in the FineTea dataset. The number of samples in the testing sets of split 1 and split 2 is not the same, so the number of instances of each action is also different. By jointly analyzing the two confusion matrices, it can be found that actions with ID values of 11, 12, 13, 14, 15, 17, and 18 achieve good recognition results. These seven actions are all green tea activities. This partly shows that the fine-grained action recognition for the green tea ceremony is relatively easy, and the fine-grained action recognition for the black tea and oolong tea ceremonies is more challenging. In terms of confused actions, action 9 is easily confused with action 2, action 21 is easily confused with action 27, and action 22 and action 29 are easily confused with each other.

## 6. Conclusions

To promote the development of action recognition methods at the fine-grained level, the FineTea dataset is constructed for fine-grained action recognition of tea ceremony actions. To better establish a fine-grained temporal model for tea ceremony actions, TSM-ConvNeXt is proposed as the benchmark method for this dataset. Extensive experiments are conducted on the FineTea and Diving48 datasets. The experimental results show that TSM-ConvNeXt obtains better experimental results than the baseline method. Compared with the state-of-the-art action recognition methods from recent years, the proposed method achieves the best experimental results with a similar parameter size. For future research, we will explore the design of lightweight fine-grained modules to improve the performance of the method in the fine-grained action recognition task. Additionally, more videos of fine-grained tea ceremony actions will be collected to expand the dataset.

## Figures and Tables

**Table 1:** Comparison of relevant datasets.

Dataset	Field	Number of Classes	Number of Clips	Video Source
MPII Cooking [2]	Cooking	67	5609	Self-collected
MLB-YouTube [3]	Baseball	9	4290	Major League Baseball
Diving48 [4]	Diving	48	18,404	Diving competition
TTStroke-21 [5]	Table tennis	21	1154	Self-collected
FineTea	Tea ceremony	31	2745	Self-collected

**Table 2:** Statistics of the FineTea dataset.

ID	Action	Number of Clips	Tea Ceremony Activity
1	Rinse the cover bowl	99	Black tea
2	Rinse the black teacup	81	Black tea
3	Present the black tea	98	Black tea
4	Place the black tea leaves	97	Black tea
5	Swirl the black teacup	88	Black tea
6	Pour out the water from the black teacup	82	Black tea
7	Infuse the black teacup with hot water	163	Black tea
8	Pour the black tea into the fair cup	136	Black tea
9	Distribute the black tea soup among the black teacups	86	Black tea
10	Serve the black tea	99	Black tea
11	Flip over the glass cup	91	Green tea
12	Rinse the glass cup	92	Green tea
13	Present the green tea	86	Green tea
14	Place the green tea leaves	90	Green tea
15	Pour water into the glass cup	79	Green tea
16	Swirl the glass cup	54	Green tea
17	Infuse the glass cup with hot water	90	Green tea
18	Serve the green tea	89	Green tea
19	Flip over the smelling cup	76	Oolong tea
20	Flip over the tasting cup	76	Oolong tea
21	Rinse the teapot	76	Oolong tea
22	Rinse the fair cup	76	Oolong tea
23	Rinse the smelling cup	76	Oolong tea
24	Rinse the tasting cup	75	Oolong tea
25	Present the oolong tea	72	Oolong tea
26	Place the oolong tea leaves	72	Oolong tea
27	Infuse the teapot with hot water	147	Oolong tea
28	Pour out the water from the tasting cup	76	Oolong tea
29	Pour the oolong tea into the fair cup	72	Oolong tea
30	Distribute the oolong tea soup among the tasting cups	74	Oolong tea
31	Serve the oolong tea	74	Oolong tea

**Table 3:** Statistics of the two sets in the FineTea dataset.

Set	Tea Ceremony Activity	Number of Clips	Total
Set 1	Green Tea	331	1378
	Black Tea	530	
	Oolong Tea	517	
Set 2	Green Tea	340	1367
	Black Tea	499	
	Oolong Tea	528	

**Table 4:** Comparison between TSM-ConvNeXt and the baseline method.

Method	Backbone	Pre-Train	Accuracy/%		
			Split 1	Split 2	Average
Baseline [17]	ResNet50	ImageNet	51.65	64.37	58.01
TSM-ConvNeXt	ConvNeXt-B	ImageNet	56.18	74.46	65.32

**Table 5:** Results of TSM-ConvNeXt method with different parameter on the FineTea dataset.

Shift Proportion Parameter	Accuracy/%		
	Split 1	Split 2	Average
1/8	56.18	74.46	65.32
1/4	59.69	76.42	68.06
1/2	45.72	67.27	56.50

**Table 6:** Ablation experiments on the FineTea dataset.

Method	Backbone	Pre-Train	Accuracy/%		
			Split 1	Split 2	Average
ConvNeXt	ConvNeXt-B	ImageNet	57.28	75.76	66.52
TSM-ConvNeXt	ConvNeXt-B	ImageNet	59.69	76.42	68.06

**Table 7:** Experimental parameters of the state-of-the-art methods.

Parameter	TimeSformer [12]	VideoSwin [13]	VideoMAE [14]	AIM [15]
Optimizer	SGD	AdamW	AdamW	AdamW
Optimizer momentum	0.9	$\beta_{1}$ = 0.9, $\beta_{2}$ = 0.999	$\beta_{1}$ = 0.9, $\beta_{2}$ = 0.999	$\beta_{1}$ = 0.9, $\beta_{2}$ = 0.999
Weight decay	1 × 10^−4^	0.05	0.05	0.05
Learning rate	5 × 10^−3^	1 × 10^−3^	5 × 10^−4^	3 × 10^−4^
Batch size	8	2	4	4

**Table 8:** Comparison with the state-of-the-art methods on the FineTea dataset.

Method	Backbone	Pre-Train	Parameter/M	Epoch	Frames	Accuracy/%		
						Split 1	Split 2	Average
TimeSformer [12]	ViT-B	ImageNet-21K	86	50	8	56.40	75.91	65.16
VideoSwin [13]	Swin-B	ImageNet	88	50	32	54.50	75.98	65.24
VideoMAE [14]	ViT-B	Kinetics-400	88	300	16	38.45	60.23	49.34
AIM [15]	ViT-B	CLIP	97	50	16	51.50	77.36	64.43
TSM-ConvNeXt8	ConvNeXt-B	ImageNet	88	50	8	59.69	76.42	68.06
TSM-ConvNeXt16	ConvNeXt-B	ImageNet	88	50	16	62.69	80.12	71.41

**Table 9:** Comparison with the state-of-the-art methods on the Diving48 dataset.

Method	Backbone	Pre-Train	Accuracy/%
CAMA-Net [40]	ResNet101	ImageNet	76.9
STC [41]	ResNet50	ImageNet	77.9
AIA(TSM) [42]	ResNet50	ImageNet	79.4
TimeSformer-L [12]	ViT-B	ImageNet	81.0
TQN [43]	S3D [44]	Kinetics-400	81.8
RPE-STDT [45]	ViT-B	ImageNet	81.8
RSANet-R50 [46]	ResNet50	ImageNet	84.2
TSM-ConvNeXt	ConvNeXt-B	ImageNet	85.5

**Table 10:** Comparison of training and testing times on split 1 of the FineTea dataset.

Method	Frames	Training Time/h	Testing Time/s	Accuracy/%
TimeSformer [12]	8	2.65	213	56.40
VideoSwin [13]	32	5.73	300	54.50
AIM [15]	16	2.08	87	51.50
TSM-ConvNeXt8	8	2.23	86	59.69
TSM-ConvNeXt16	16	3.45	129	62.69

## Data Availability

The data that support the findings of this study are available from the corresponding author upon reasonable request.
